# Using Bayesian Nonparametric Hidden Semi-Markov Models to Disentangle Affect Processes during Marital Interaction

**DOI:** 10.1371/journal.pone.0155706

**Published:** 2016-05-17

**Authors:** William A. Griffin, Xun Li

**Affiliations:** 1 Center for Social Dynamics and Complexity, ISTB-1, 530 East Orange Street, PO Box 874804, Arizona State University, Tempe, 85287-4804, Arizona, United States of America; 2 Sanford School of Social and Family Dynamics, Arizona State University, Tempe, Arizona, United States of America; 3 GeoDa Center for Geospatial Analysis and Computation, School of Geographical Sciences and Urban Planning, Arizona State University, Tempe, Arizona, United States of America; University of Manchester, UNITED KINGDOM

## Abstract

Sequential affect dynamics generated during the interaction of intimate dyads, such as married couples, are associated with a cascade of effects—some good and some bad—on each partner, close family members, and other social contacts. Although the effects are well documented, the probabilistic structures associated with micro-social processes connected to the varied outcomes remain enigmatic. Using extant data we developed a method of classifying and subsequently generating couple dynamics using a Hierarchical Dirichlet Process Hidden semi-Markov Model (HDP-HSMM). Our findings indicate that several key aspects of existing models of marital interaction are inadequate: affect state emissions and their durations, along with the expected variability differences between distressed and nondistressed couples are present but highly nuanced; and most surprisingly, heterogeneity among highly satisfied couples necessitate that they be divided into subgroups. We review how this unsupervised learning technique generates plausible dyadic sequences that are sensitive to relationship quality and provide a natural mechanism for computational models of behavioral and affective micro-social processes.

## Introduction

Decades of marital interaction research demonstrate an unequivocal relationship between moment-to-moment behavioral exchanges seen in a conversing couple and the quality of their relationship: each gesture and word is cradled in affect; each nuanced pause conveys a semi-private message understood only to the inhabitants of the relationship and the entire process is uniquely sensitive to the situation. The language is public but the conversation is private. And despite almost forty years of investigators dissecting sequential verbal and non-verbal data, the micro-social processes that critically determine and reflect marital quality remain opaque.

Social scientists studying the dynamics of ubiquitous self-organizing dyadic processes—either as family units or merely actors in a fecundity play—have little insight into the critical features that predict, or even describe, sustained coupling. For humans, this is critically important: Evidence suggests that the marital relationship has unequivocal effects on health—a good one protects and buffers its constituents whereas bad one is associated with increased risks for physical maladies [1, 2], along with increases in drug and alcohol use, reported depression, and job instability. These effects extend beyond the dyad—a distressed marriage has a corrosive effect on parent-child relationships and increases psychological problems in children. Should the relationship end, neither the children or the adults are immune, each endures greater stress and accompanying decreases in health and financial stability [3].

Since observational studies of marital interaction began in the mid-1970s, investigators had hoped to establish a set of critical macro-level behavior patterns, derived from micro-social processes, that predict marital quality beyond the observed constituent micro-social behaviors (e.g., negative begets negative) [4]. Collectively these studies show that although each dyad is unique, when aggregated across differing levels of self-report marital satisfaction, they show consistent, albeit slight, structural differences at each level of aggregation. These composite structures, using the prevalent categorical coding systems of the time—composed of verbal statements, behaviors and affect—uniquely characterize within group satisfaction levels. Most of these studies sought to illustrate how sequential couple behavior integrates with affect. Emphasis was on analyzing, illustrating, and classifying one or two modalities of behavior (e.g., nonverbal) that effectively discriminates couple type by satisfaction level. To date, however, only two process features reliably, albeit weakly, signify marital distress: greater negativity and longer chains of negative reciprocity; aside from these, no summary or sequential features of a dyad interacting consistently reflect marital quality, and none foretell marital outcome [5].

In effect, and not surprisingly, these findings suggest that marital quality determines the affective and behavioral generating process which, in turn, instantiates the observed dyadic behaviors. At this point, however, it’s unknown how the constituent parts—temporal and behavioral—of the generating process are interrelated or arranged (i.e., its topology); nor has there been any attempt to articulate a catholic analytic solution allowing investigators to reanalyze extant data, with the hope of uncovering additional crucial tempo-behavioral facets linked to self-report satisfaction in intimate dyads. This deficient exists, in large part, due to the lack of appropriate analytic tools.

Over the last few decades most of the significant insights into couple dynamics arose not from better data or measurement methods but improved data analytic strategies [6]: the area moved steadily from summary statistics describing individual behavior during an interaction (e.g., mean number of positive statements) to probability estimates of shifting dyadic structures (e.g., sequential analysis [7]) to state transition models (e.g., Hidden Markov Models [8]).

Despite these analytic advances, it’s commonly acknowledged that the algorithms that generate the evolving contingency structures embedded in sequential data are not well understood [5]; in turn, this has severely limited attempts to model dyadic interaction (cf. [9] or more recently, [6] [mother-child interactions]). Even with the abundance of real-time couple interaction data from multiple labs throughout the world there are no published generative models of dyad dynamics and certainty none that discriminate by relationship satisfaction level. To construct realistic and informative generative models of dyadic interactions three analytic problems need addressing: (1) how to articulate the state space of the dyad; (2) how to generate a tenable state transition matrix; and (3) how to incorporate duration expectancy into states and transitions.

The emerging field of Affective Computing offers a glimpse of how to build these generative models. Affective Computing focuses on, by combining computer science and engineering, methods of generating plausible affect associated with social interaction for the purpose of building realistic robots and avatars, as well as constructing better algorithms and computational models of affect, its expression and use, in social dynamics [10, 11]. In practice, Affective Computing is a scientific chimera that integrates existing literature on verbal and non-verbal social behavior, such as that cited above, incorporates ongoing laboratory studies on affect (e.g., computer-user interfaces) and builds computational models using methods drawn from the machine learning field [12]. It’s this latter ability that is lacking in the existing marital interaction literature—reinvigorating this area requires moving from the language of social science and into the realm of computer science: specifically, to advance our understanding of intimate dyadic affective and behavioral structures we need generative models of sequential latent processes, i.e., profiles of sequential movement across latent states with estimated probabilistic structures [13].

Fortunately over the last decade analyzing latent generating processes became a cornerstone of contemporary machine learning theory [14]. With the advent of faster computers, and theoretical advances in Bayesian methods, emphasis shifted toward discovering methods that capture temporal clustering and feature extraction in sequential data. At the forefront of this area is the family of nonparametric Bayesian techniques. For example, Hierarchical Dirichlet Process methods permit investigators to partition data into topics [15] and sequence sensitive feature states [16]. Likewise, renewed interest in the traditional Hidden Markov Model (HMM) has resulted in several revised multi-state, hierarchical models having the ability to capture time-sensitive latent state transition processes (see e.g., infinite HMM [17, 18]; Hierarchical Dirichlet Process-HMM (HDP-HMM), [15, 19]).

Initially, HMMs were used as tools for voice recognition [20] and only later adopted for myriad recognition tasks, ranging from social dynamics [8] and biological sequences [21] to economic and hydrological time-series [22]. Recently HMM methodologies began incorporating the capacity of nonparametric Bayesian approaches to define prior distributions on transition matrices over countably infinite state spaces; adopting this technique allows a greater range of use with real, somewhat messy, data [23, 24]. Contemporaneously, Fox and colleagues [23] began developing algorithms that parameterize the likelihood of state self-transitions; this, combined with the use of a Hierarchical Dirichlet process to generate priors while leaving unspecified the expected number of states, permits the modeling of duration sensitive latent state transitions (i.e., semi-Markovian processes). Such models are more realistic of natural dynamic stochastic processes [25].

Borrowing heavily from the innovations of Fox and colleagues [23] along with the recent work by Johnson and Willsky [24], we use a explicit-duration HDP-HSMM of couple affect dynamics to build generative models illustrating differential affect patterns in marital couples classified by self-report satisfaction. This paper illustrates how this methodology can capture pertinent sequential dyadic state dynamics and accurately discriminate affective processes associated with relationship satisfaction.

### Preliminary Work

Almost a decade ago Griffin [8] used dyadic affect sequences from 30 couples to develop a 10-state 4-symbol HMM that classified distressed from non-distressed couples with an accuracy rate of 91%; he found a decidedly different distribution in the observables for self-transitions and states transitions among maritally satisfied couples—there was substantially greater mutual positive affect during the middle phase of the sequence. Three aspects of this research were noteworthy. First, the classification rate of 91% is below expectation in a well developed HMM, but this value is acceptable, especially in this social science area, given the small sample size and low dimensional vector used to create the data string. Second, although no substantive conclusions were forwarded, results demonstrated that couple interactions were patterned and that accurate machine learning and classification can occur without supervision. And third, when these analyses occurred, Hidden semi-Markov Models, although well articulated in theory and few examples were in the literature, practical implementation was years away [25].

Although a decade ago HMMs were well developed mathematically, their traditional use required the investigator to assume time-invariant geometric state distributions and a fixed known number of states. In a dynamical system, by definition, time in state is a critical feature; likewise, accurate descriptions and models of different systems require differing number of states. Finally, the original investigation divided the couples into non-distressed and distressed, and although consistent with literature, this simple dichotomy constrained the ability to explore the subtle behavioral differences that exist among couples expressing a diverse range of marital satisfaction levels.

Fortunately, with increased investigations of real-world complex datasets, the standard HMM has been transformed over the last decade—computer scientists created numerous sequential analytic techniques that are sensitive to the nuances of evolving latent structures (e.g., infinite HMMs) akin to the type seen in micro-social dynamics [14]. Among these is the HDP-HMM [15]. The hierarchical Dirichlet process (HDP)–a nonparametric technique using the Dirichlet process (i.e., a distribution over distributions)–models the dependence among groups through sharing the same set of discrete parameters. Yet its assumption of exchangeability make it inappropriate for sequential data [26]; fortunately this restrictive assumption spurred new models that are appropriate for time sensitive data [13, 27].

For example, Fox, Willsky, and colleagues [23] were able to extend the standard HDP by introducing a method of parameterizing the self-transition bias, the state persistence problem, which in turn, allowed them to develop a fully nonparametric HMM—effectively removing the need to specify, a priori, the number of states associated with a system. This method, termed the Sticky HDP-HMM, despite being a radical improvement, suffered the same duration distribution constraint as the standard HMM: state durations are time-invariant. Fortunately Johnson and Willsky [24] quickly extended the work of Fox et al. [23] by combining semi-Markovian ideas with the Sticky HDP-HMM to construct a general class of models that incorporated duration distributions. The recent models are well described in the aforementioned references. A methodological overview of these techniques is given in S1 Appendix.

The present work incorporates the methodology developed by Johnson and Willsky; our goal of extracting patterns from sequential data is conceptually and quantitatively similar to their search for structure in real and synthetic data. Whereas they used multiple time-series of household appliance data, we inserted husband and wife sequence data, and we have added the additional dimension of relationship satisfaction as a classification problem. Additionally, our simulations are written in Python, as is their publicly available code, thus it was modified to fit our research questions, output plots, and GUI development.

Theoretical models of real-time observable dyadic processes, especially marital dynamics as well as parent-child or mother-infant interactions, ceased evolving over a decade ago [5]; this epoch of stagnation resulted from at least two factors: (1) increasing costs made collecting lab based observation data very expensive; (2) sequential data from behavioral interaction labs used data analytic methods that, although state-of-the-art at the time [7], severely lag behind contemporary machine—learning techniques that analytically integrate event duration and pattern recognition methods [28, 29]. Likewise, Affective Computing, a scientific area at the forefront of computational modeling of social processes, lacks representative models of intimate relationships—that is, paired individuals who have a history of interactions where the affective exchanges have unique meanings within the context of the couple and their history. Thus for this study we show that integrating temporal and event data with modern machine learning—techniques provides a novel way of capturing the nuances of sequential dyadic data that differentiate couples by levels of satisfaction and provide the foundation for building robust computational models in both areas.

## Materials and Methods

**Fig 1:**
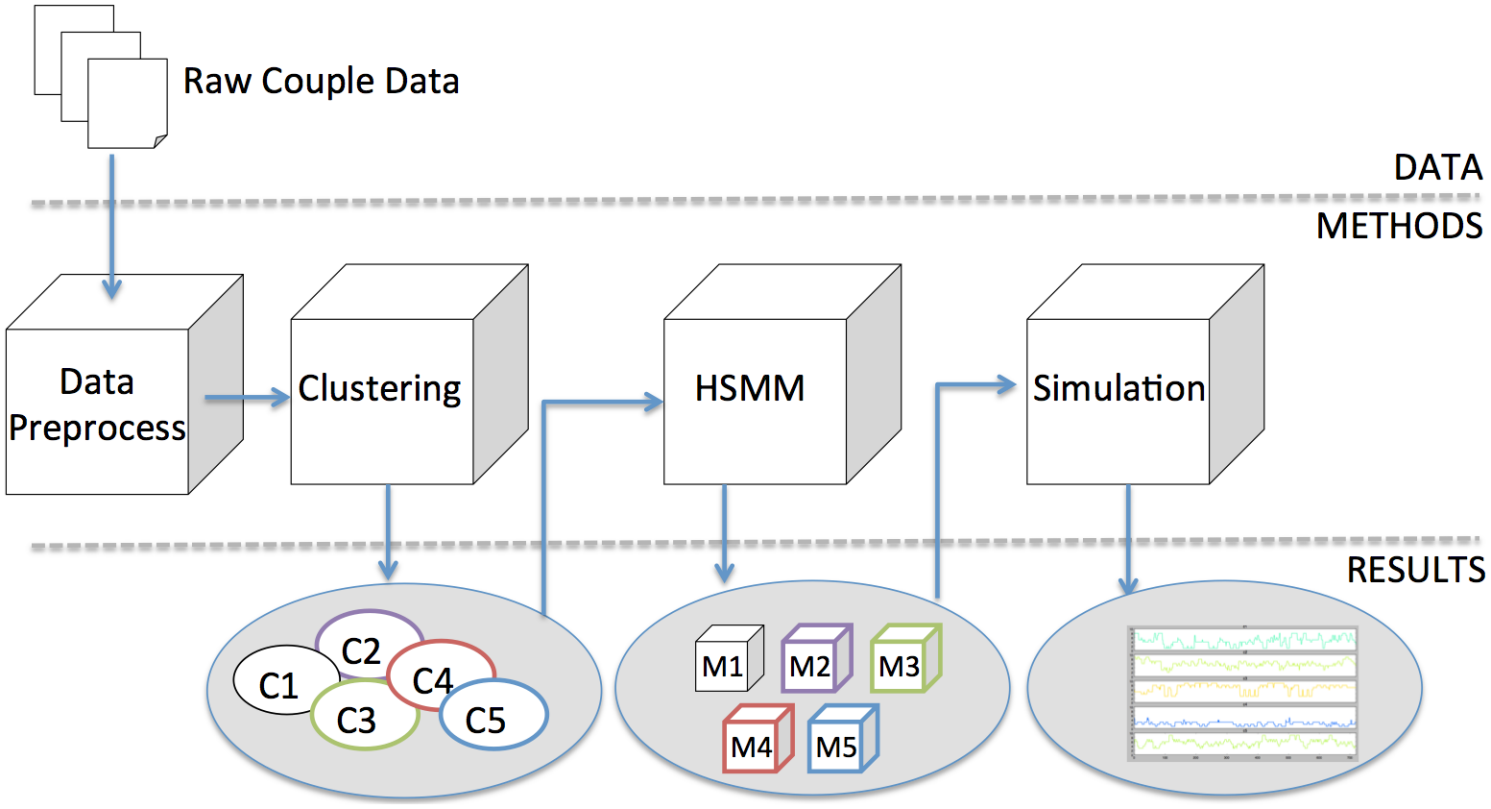
Process Flow Of Data Preparation, Modeling, and Simulation.

To use the HDP-HSMM methodology required that we partition the couples into homogeneous sub-groups. To do this, we moved away from the traditional reliance on self-report data and developed a dyad feature set which allowed us to use hierarchical clustering techniques to construct the sub-groups. Using a data driven feature set for generating sub-groups among marital dyads is novel; to clarify how this was done we have included a description of the clustering process in this *Methods* section, and in addition, because of the added interpretative value the clustering provides, we added a *Clustering* component to the *Results* section. An overview of data processing scheme encompassing the extant data, creating sub-groups and then modeling the derived clusters followed by simulations is shown in Fig 1.

### Sample and Recruitment

During 1992-1993 thirty married couples responded to newspaper advertisements offering twenty-five dollars for participating in a study about marital communication. Participants were recruited from among married couples living in a large metropolitan area in the Southwestern United States. In the first wave, 38 participants (19 couples) were recruited; five of the 19 couples reported marital distress based on a standard marital satisfaction questionnaire [30]. A subsequent modified newspaper advertisement asked couples to participate in the study if they felt their marriages were distressed; of the 11 married couples recruited in the second wave, two were nondistressed and nine qualified as distressed; in aggregate, 14 distressed and 16 nondistressed married couples are used in the current study.

Couples in the study were typical of those found in marital interaction studies: relatively young and moderately educated lower-middle-class couples with one child at home. A series of t-tests found that, other than the number of children in the home (*t* (28) = 2.10, p < .05, *df* = 28; distressed = 1.42, nondistressed = .81), the groups were not statistically significantly different on salient demographic features. Eighty percent (24) of the couples were Caucasian, 16 percent (5) were mixed ethnically, and one was African-American. See [31, 32] for additional recruitment and demographic information. Prior to commencing the study, the data collection procedure along with all questionnaires including consent forms and data storage plans were submitted to and approved by the Arizona State University (USA) Human Subjects Internal Review Board. All participants signed the approved consent forms detailing rights of withdrawal along with the constraints on data usage and storage. All data are maintained without identifying information and available via request to the corresponding author. Additional social and demographic information in provided in S1 Text.

### Laboratory Procedure

After arriving at the university Marital Interaction Lab, couples were seated in a room constructed to resemble a small living area containing wall prints, curtains, plants, and two chairs in the center of the room. Two unobtrusive, partially concealed, remotely controlled cameras were mounted on the walls at head level behind each chair. All audio-visual and mixing equipment was controlled from a room adjacent to the interaction. Video signals were combined producing a split screen image with audio being obtained from lavaliere microphones worn by each spouse.

### Problem Solving Task

After completing informed consent forms, couples were given the Areas of Disagreement Inventory [33]. From a list of typical areas of conflict in a marriage, each marital partner selected and ranked three topics that he or she thought was most problematic in their relationship. With the help of a lab assistant the couples then negotiated on the top three areas from their joint list. Prior to beginning an interaction based on these topics the couple moved to separate section of the laboratory and was shown how to use the affect rating software (see below). After becoming familiar with the software, they returned to their chairs and the lab assistant, prior to leaving the room, instructed the couple to attempt to resolve the topics while engaging in a 12-minute discussion. This is a common task used to evoke relevant interaction in married dyads [34]. After leaving the room, a small red light indicated to the couple to commence their discussion; after 12 minutes the light was turned off and each dyad member separated and independently rated the interaction (see Affect Rating).

### Affect Rating

Spouses were separated immediately after the conversation and taken to another section of the lab where each individual simultaneously rated his or her own affect during the interaction while viewing a split-screen playback. Separated by a large solid partition and wearing audio headsets, husbands and wives could not see or hear their spouse while reviewing the videotape.

Video feed was played back through a specially configured computer using software that overlays a 9-level, color-coded, vertical bar on the 19-inch color video monitor. This overlay was positioned beside the face on the monitor of the individual reviewing the video. Affect ratings ranged from extreme negative (red), through neutral (gray) to extreme positive (blue), and was controlled by a pc mouse. Extreme negative was at the monitor bottom, neutral at mid-monitor, and positive at the top of the monitor; bar width varied at each affect level (5 pixel increments) corresponding to the intensity of the affect, neutral being the thinnest; at its widest—extreme negative and positive—the bar was 28 pixels wide (1.5 cm). As the reviewer moved the mouse, the affect bar was high-lighted corresponding to the degree and direction of the affect. Prior to reviewing the video, and viewing only his or her own rating, each spouse was asked to move the mouse to reflect affect experience during the interaction (i.e., “How were you feeling at each moment?”). Software recorded the location of the bar position every second, providing a continuous measure of affect throughout the interaction. The assistant left the room as the playback began; to insure participant compliance, the reviewing process was monitored in the adjacent equipment control room. Validity for this procedure was established by Gottman and Levenson in 1985 by comparing participant ratings to observational coding [35].

In this method of metric retrieval, each affect has a subjective reference that is unique to the rater, within the context of the interaction, given the dyad’s history. For each individual there is only an internal template referencing their affective state; an internal state that is pleasant to one individual may be only neutral to another. Moreover, because it is self-report, it arguable that such a recall procedure provides a good proxy of the true affect state, and requires less inference than other, outsider perspective data collection procedures. This method of assessing affect effectively discriminates, by sex, the propensity to exit negative states [31], and Griffin [8], using Hidden Markov Models, found that affect ratings and their durations successfully discriminated distressed and nondistressed couples.

During the 12-minute interaction each couple produced approximately 720 seconds of affect recording (*μ* = 717; variation due to equipment error). General descriptive moments of the original 9–point scale show the expected tendencies: distressed couples expressed more negative affect (*μ* = 4.79, *σ* = 1.05) than the nondistressed couples (*μ* = 3.76, *σ* = 1.37), *t*(28) = 3.27, p < .000 (one-tailed) and within sex across group ratings were also significantly different; males (distressed *μ* = 4.56, *σ* = .91, nondistressed *μ* = 3.7, *σ* = 1.41): *t*(28) = 1.98, p < .02 (one-tailed); females (distressed *μ* = 5.03, *σ* = .1.16, nondistressed *μ* = 3.82, *σ* = 1.38): *t*(28) = 2.59, p < .001. Within group across sex differences were not significant.

### States Construction

Inclusion of self-report affect as a dyadic feature is relatively straightforward: we assume, based on ample evidence, that happily married couples interact differently than those who are dissatisfied [5]. This suggests that dyads, of any type or configuration, can be categorized by their qualitative state, and the criteria for categorizing would be based on measurable characteristics observable during intra—dyadic interactions [36]. Assuming that the feelings of one spouse toward their partner is generated by an attribution set—a cognitive structure that evolved during the couple’s history and is maintained by current events—it follows that the internal feelings manifested by the attribution set are expressed as observable verbal statements and nonverbal behaviors along with self-report affect [37].

**Fig 2:**
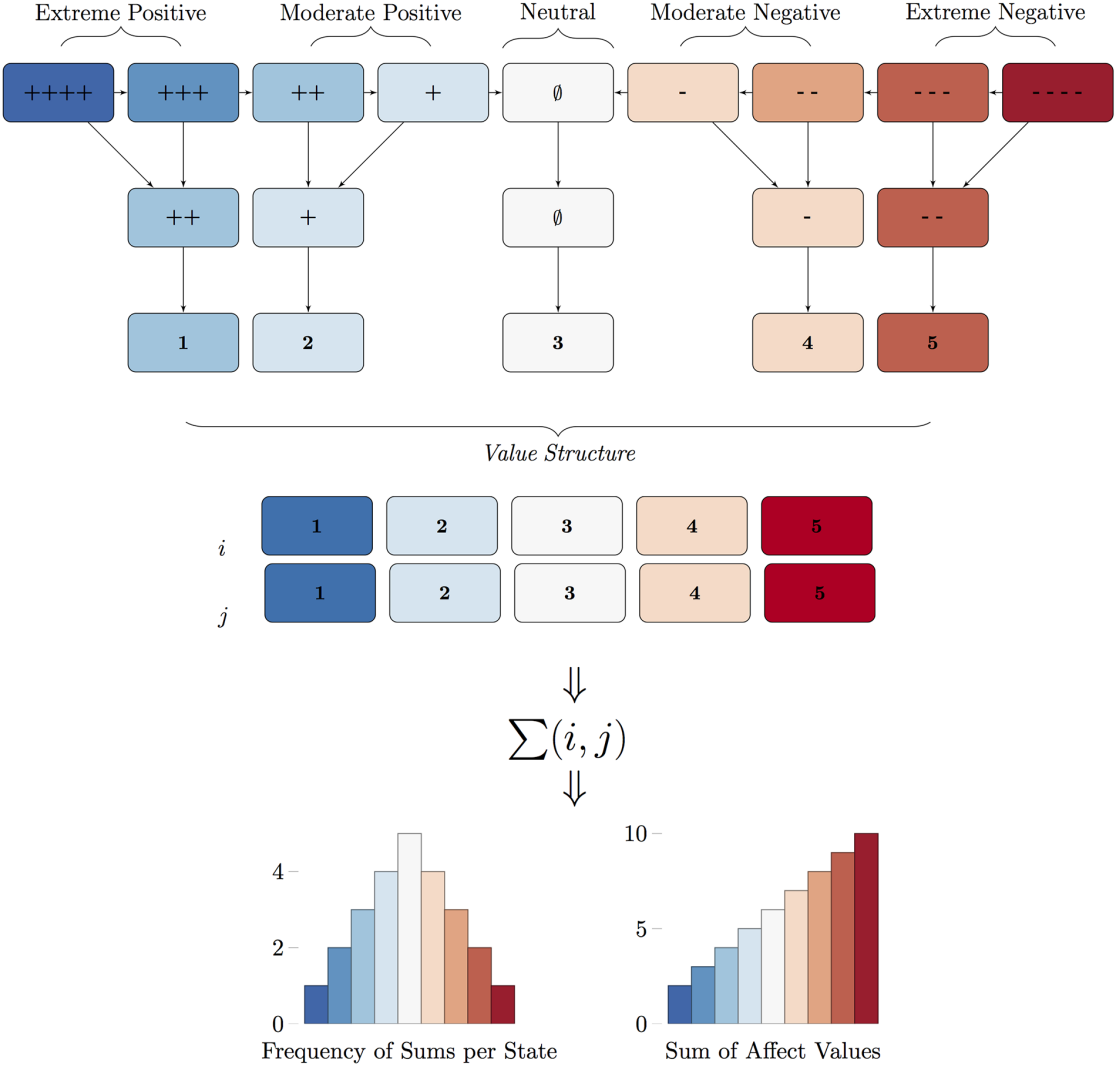
States Data Reduction Scheme. Original categories are shown at the top; these were collapsed and summed within dyad. Profiles of possible frequencies and summed values are shown at the bottom, left and right, respectively.

In the original data, individual affect ranged from 0 (extreme positive) to 8 (extreme negative); these values where then converted to a 5-point scale to ease interpretation: 1-extreme positive; 2-positive; 3-neutral; 4-negative; 5-extreme negative. Next, the sum of the joint affects were used to create a couple States score. Generating States this way assumes that the States variable is an estimate of the proximal relationship quality of the couple, being composed of his and her joint state; the sum indicates the overall condition expressed as a single state. The States range from 1-9, lower values reflect greater positive affect. The States construction scheme is shown in Fig 2. In this configuration, States, being comprised of multiple discrete states, forms an ordered multinomial distribution.

### Clustering

Historically couples were classified as either distressed or non-distressed according to their joint score on one of several self-report marital satisfaction instruments, the most common being the Marital Adjustment Test (MAT; [30]; see Methods section for an overview). Although most investigators knew that a simple cut-score (MAT > 100) was a crude way to discriminate distressed marriages from those that professed higher levels of satisfaction, nonetheless most data samples were too small to be analyzed on a continuum. At best, with modestly large samples, it was possible to divide the couples into quantiles, but even then distributing couples into discrete groups based on continuous scores was mostly for intuitive convenience and not on known feature differences. Once placed into categories, investigators tried to find differences in affect, along with verbal and nonverbal behaviors, among the classified groups (see [34]for an overview of this work). Likewise, we initially used the traditional MAT > 100 score to separate the couples into distressed and non-distressed groups; with some preliminary analyzes it was immediately clear that this simple split contained heterogeneous affect distribution subgroups. Next we split the couples into (*L*ow, *M*edium, *H*igh) tertiles; cut-points were *L* < 95;< *M* <;117.5 < *H*. Again it became obvious that within group heterogeneity, at least relative to affect ratings, made substantive interpretations about the appropriate category impossible. We then decided to create a multi-dimensional feature set to cluster and classify couples.

**Feature Set** Moving away from the traditional one source method of classifying couples we constructed a 5-dimension feature set derived from three sources: (1) the States variable–(a) *Shannon entropy*, (b) *mean*, (c) *standard deviation*; (2) intradyadic, cross-sex, affect expression similarity–(d) *dynamic time warping* (DTW); and (3) (e) self-reported *marital satisfaction*.

**Entropy Estimates** From the States variable we derived a Shannon entropy estimate [38]. Entropy in this circumstance reflects the dyad’s affect distribution given the local context (i.e., conversation, behaviors observed, etc.) and distal sentiment about the relationship [37]; we assume that proximal and distal influences are not exclusive of each other. Comparing Shannon entropies among couples requires that their state space be equal; to construct equivalent spaces we used the Bayesian technique of regularizing the cell counts by converting the States distribution into a Dirichlet multinomial distribution using Jeffreys prior [39] and the Krichevsky-Trofimov estimator [40, 41]. Estimates were generated using the R program *entropy*[42]. States entropies ranged from 1.324–3.041 (*μ* = 2.382, *σ* = 0.412).

**Fig 3:**
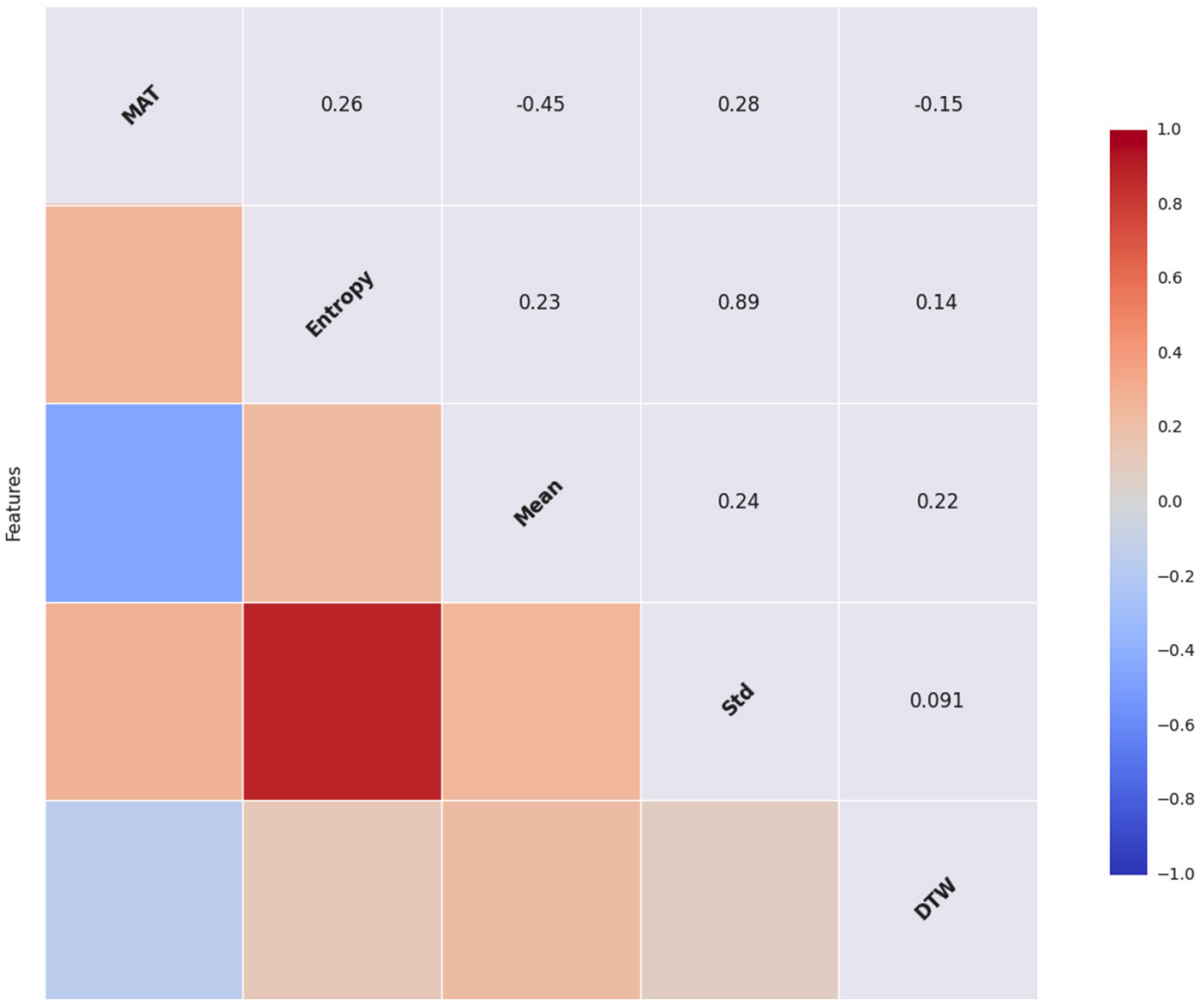
Feature Set Correlation Matrix. MAT refers to marital satisfaction; Mean refers to States mean; Std refers to States standard deviation.

**Means & Standard Deviations** Two additional features were also taken from the States variable; the first is the average States score, values ranged from 2.001 to 7.467 (*μ* = 5.336, *σ* = 1.463); lower scores indicate greater positive affect. Second, we used the States standard deviation; values ranged from 0.654 to 2.282 (*μ* = 1.479, *σ* = 0.445). Inter-feature correlations are shown in Fig 3; note that, not surprisingly, entropy and the States standard deviation are highly correlated, others are in the expected direction and range.

**Dynamic Time Warping** A feature of any intradyadic interaction is the discrepancy of relevant behaviors between the interactants—in these data it’s affect rating. Rather than take a simple distance measure we used Dynamic Time Warping (DTW) [43, 44] to estimate the cost of aligning the affect sequence values. We used the raw male and female reduced 5-point scale ratings. Estimates of the DTW were done with the R program *dtw* [43]. Higher values indicate greater discrepancy between the interactant’s affect ratings. Dyad level dynamic time warping values ranged from 24 to 991 (*μ* = 291.633, *σ* = 191.883).

**Marital Adjustment Test** Self-report marital satisfaction is typically assessed using a few well established inventories, among these is the Marital Adjustment Test (MAT) [30]; it is a widely used measure of each spouse’s degree of marital satisfaction [45] and has the greatest number of reliability and validity studies of all self-report marital adjustment measures [46]. Reliability coefficients for the MAT range from .73 to .90 [30]; internal consistency is .83 [47]. The MAT is a Likert—type instrument that enables researchers to discriminate distressed from nondistressed married couples; scores can range from 2 to 158, the extremes of distress and nondistress, respectively. The couple score is the average of the husband and wife score. A score of 101 or greater indicates a nondistressed marital dyad and a score less than 101 indicates marital distress. For this sample, item analysis using Cronbach’s alpha showed moderate and adequate internal consistency; .75 across the entire sample of males and females, .79 for all males, and .73 for all females. Participants in the current study had couple MAT scores ranging from 44 to 138 (*μ* = 101.433, *σ* = 25.903).

Consistent with the current practice of initially examining the differences by distress level, within group across sex differences in reported marital satisfaction (MAT) was not significant in either group according to the results of paired t-tests. As expected, the groups differed by marital satisfaction whether assessing within sex (husbands: (*t*(28) = 7.06, p < .001; wives: *t*(28) = 5.80, p < .001) or averaged at the couple level (*t*(28) = 7.87, p < 001.

### The Hierarchical Dirichlet Process Hidden semi-Markov Model (HDP-HSMM)

### Parameter Setup

For analyzing dyadic interaction, either, e.g., mother-child or spousal interactions, the capabilities of the explicit-duration HDP-HSMM extends the traditional Hidden Markov Model in two fundamental ways: (1) by incorporating varying state durations, micro-social event dynamics are not constrained to a geometric form—acknowledging that time in state makes a difference in sequential behavior; and (2) by allowing a countably infinite number of states the model incorporates dyadic histories with ideographic state spaces—each dyad has a unique number of states that best capture their behavioral propensities. To incorporate duration distributions and transitions likelihoods the models described herein used four basic distributions: observations, durations, states, and transitions [23, 24]. See S1 Appendix for an overview of these methods. In typical nonparametric Bayesian modeling fashion, each was generated with a conjugate prior using hyperparameters [19, 23]. Of these, Observations and Durations used informed priors.

**Observations** Observations (i.e., affect ratings) were modeled using a continuous Gaussian distribution. To generate the Gaussian distributions we used its conjugate prior—the normal-inverse-Wishart distribution, a multivariate four-parameter (*μ* = prior mean, *λ* = scale matrix, *κ* = prior observations, *ν* = degrees of freedom) family of continuous probability distributions. Of the four hyperparameters, two have only minimal effects on the model and are set during initialization: *κ* = .25, and *ν* = 4, where *ν* represents the number of dimensions (i.e., male and female affect ratings) * 2; the other two, *μ* and *λ* (covariance), were generated from realized data for each cluster.

**Durations** State durations were modeled as Poisson distributed, with parameter *λ*; appropriate parameters were estimated using hyperparameters (*κ* = shape, *θ* = scale) for a Gamma conjugate prior. Using the durations of each States state, the gamma parameters for each cluster were estimated by using L-moments [48] from realized data.

**States** A single parameter Dirichlet Process conjugate prior using an initial concentration parameter *α* generated the multinomial distribution of states.

**Transitions** State transition distributions were represented as a Hierarchical Dirichlet Process (HDP) across couples. Initial construction of the HDP model was controlled by 2 hyperparameters: *α* and *γ*; where *α* represented the total mass concentration for each row of the transition matrix and *γ* was the hyperparameter used in the stick breaking distribution (denoted as GEM) to generate the random base measure [49]. At each couple level the transition matrix was generated as a Dirichlet Process, where collectively, they were tied together via the HDP through *β*, where *β* ∼ GEM(*γ*).

### Model Selection

Models were selected by minimizing the difference between the realized and simulated data. Specially, model selection was done by comparing and selecting the best fitting model derived from 50,000 Gibbs samples over a parameter sweep of values that initial analyzes suggested were most sensitive to model fit; these were: Transitions: *α* {4, 8} and *γ* {4, 8}; Maximum state duration {30, 60, 90}; and Maximum number of states {15, 20}. Each parameter sweep consisted of 24 separate runs (*Transitions*[*α*](2) *Transitions[*γ*](2)*Duration(3)*MaxStates(2)), each with 10,000 Gibbs samples for all 30 couples; this was done for 5 independent waves of analysis for each Cluster. We did not assume that clusters required similar models. It was possible therefore to have 5 *best* models, one for each cluster. Alternatively, it was possible that each individual dyad would have a best fit irrespective of which cluster they was assigned.

Model selection was determined by assessing sequence dissimilarity between the model simulated sequence and the raw data for each couple within a cluster; best fit was derived by averaging the 5 waves of each parameter configuration and the combination with the smallest average difference was considered the best model. We used the States values for comparison. Comparisons were done using TraMineR [50], an R based tool that estimates differences between sequential categorical data. We used two distance estimators: Hamming distance and Dynamic Hamming Distance (DHD); this latter method estimates distance by uncovering contemporaneous similarities and generating substitution costs as a function of transition densities [51].

## Results

### Clusters

Each feature (*MAT Score*, *States mean*, *States standard deviation*, *Shannon entropy*, *Dynamic Time Warping*) was initially normalized (0,1) using the MinMax method. After normalizing the data, a series of pair-wise euclidean distance estimates were taken (row 1 vs row 2, etc). This normalized distance matrix was then used to construct clusters.

To generate clusters we employed hierarchical clustering techniques, alternating between average linkage and Ward’s method [52]; the eventual number of clusters varied between 4 and 5, and both techniques produced similar, although not identical clusters. The average linkage method with 5 clusters was slightly different than the Ward’s method for a single couple; it left a singleton whereas the same dyad was inserted into an existing cluster using the Ward’s method. We finally settled on the 5-cluster model using Ward’s method [53].

The final clusters varied in sample size from 3 couples (Cluster 4) to 10 (Cluster 2), and in reference to marital satisfaction, the final clusters represented 1 low (Cluster 5), 2 medium (Clusters 2,3), and 2 high satisfaction groupings (Clusters 1,4). Several methods, intuitively and statistically, were used to verify the validity of the clusters.

Because of the small and varied sizes for the number of couples comprising the clusters, traditional statistical methods were not appropriate; however, it was possible to use the Bayesian BEST method to compare aggregate raw States values by cluster [54, 55]. A Bayesian analog to the traditional parametric two—sample t-test, BEST estimates the difference in means between two groups and yields a probability distribution over the difference. None of the States pairwise cluster comparisons spanned 0, indicating significant differences within each pairing.

**Fig 4:**
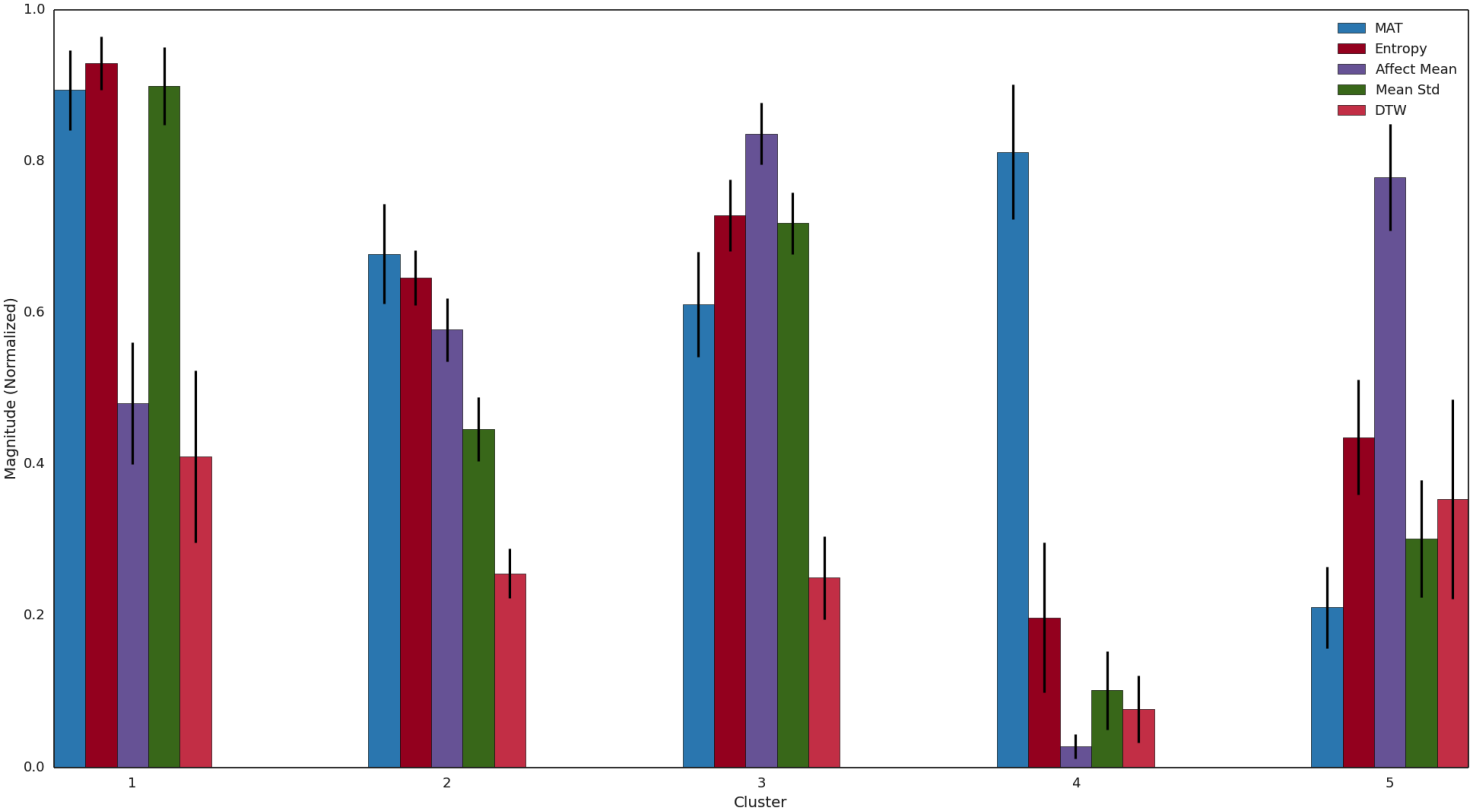
Distribution of Normalized Features. MAT refers to reported marital satisfaction; States Mean refers to joint affect expression. MAT and Mean are inversely scales.

Examining Fig 4 shows how the Clusters differed in the five couple normalized feature domains. Its evident that Clusters 1 and 4 are the high self-reported marital satisfaction groups (MAT) whereas Cluster 5 has the lowest satisfaction score. Moreover, the two high satisfaction Clusters differ significantly in their Mean Affect Scores (i.e., for States, lower values indicate more positive affect); in addition, the standard deviation of the affect was minimal in Cluster 4 whereas Cluster 1 had the highest among all Clusters. Finally, note that the DTW estimate is lowest in Cluster 4 and highest in Cluster 1. Thus, among all clusters, the highest discrepancy in computational cost of matching the male and female affects is greatest between high satisfaction subgroups. In essence, these data suggest there is at least two types of interaction among highly satisfied couples.

**Fig 5:**
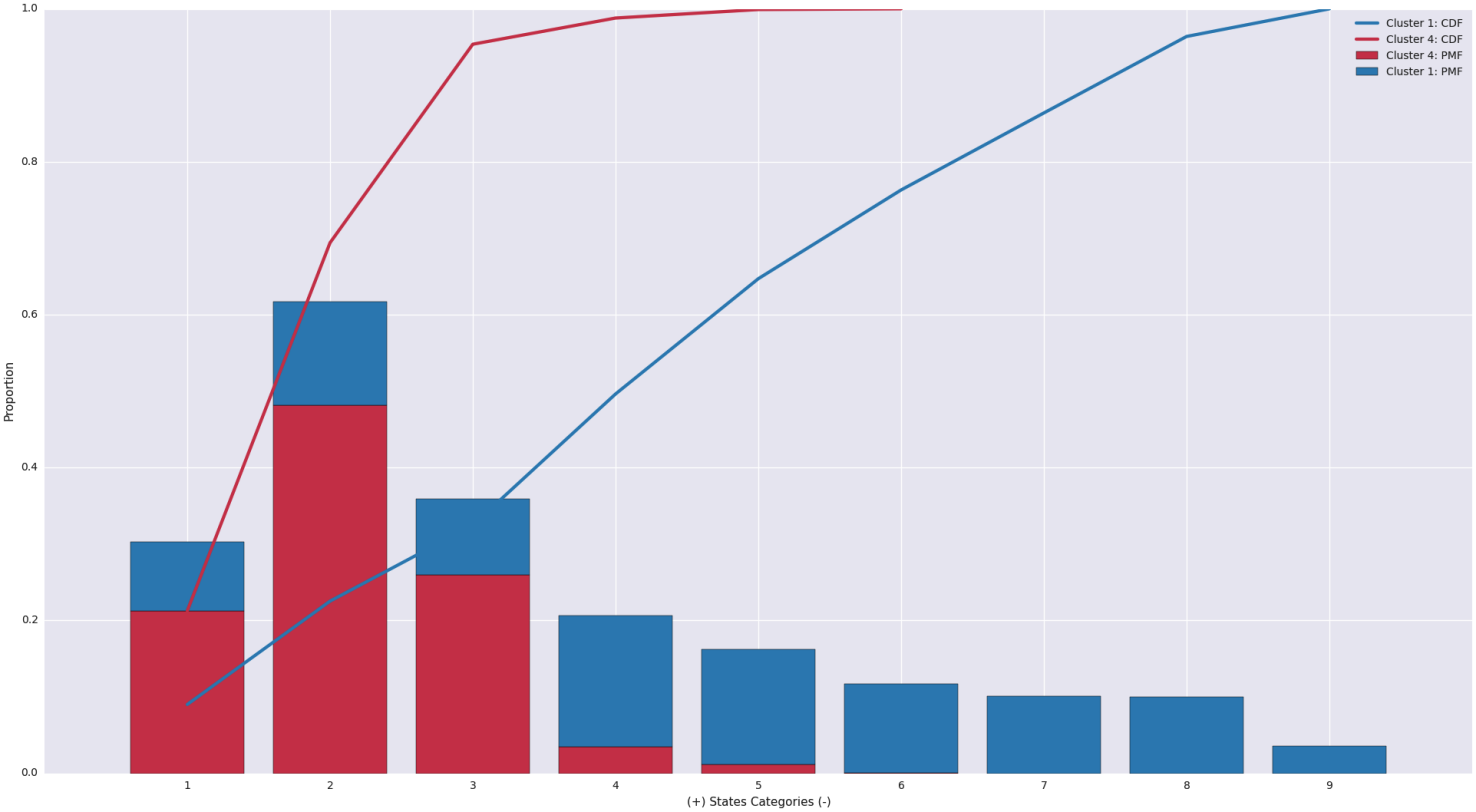
Stacked bars showing PMF and CDF of Clusters 1 and 4. States distributions indicate that unlike the couples in Cluster 4, Cluster 1 couples distribute affect across the range of States.

Simply plotting the respective States distributions show a clear distinction between these two clusters (see Fig 5). By overlaying the cumulative density function (CDF) with the probability mass function (PMF), it’s evidence that Cluster 1 disperses affect across the possible range whereas Cluster 4 is constrained to the most positive States.

**Fig 6:**
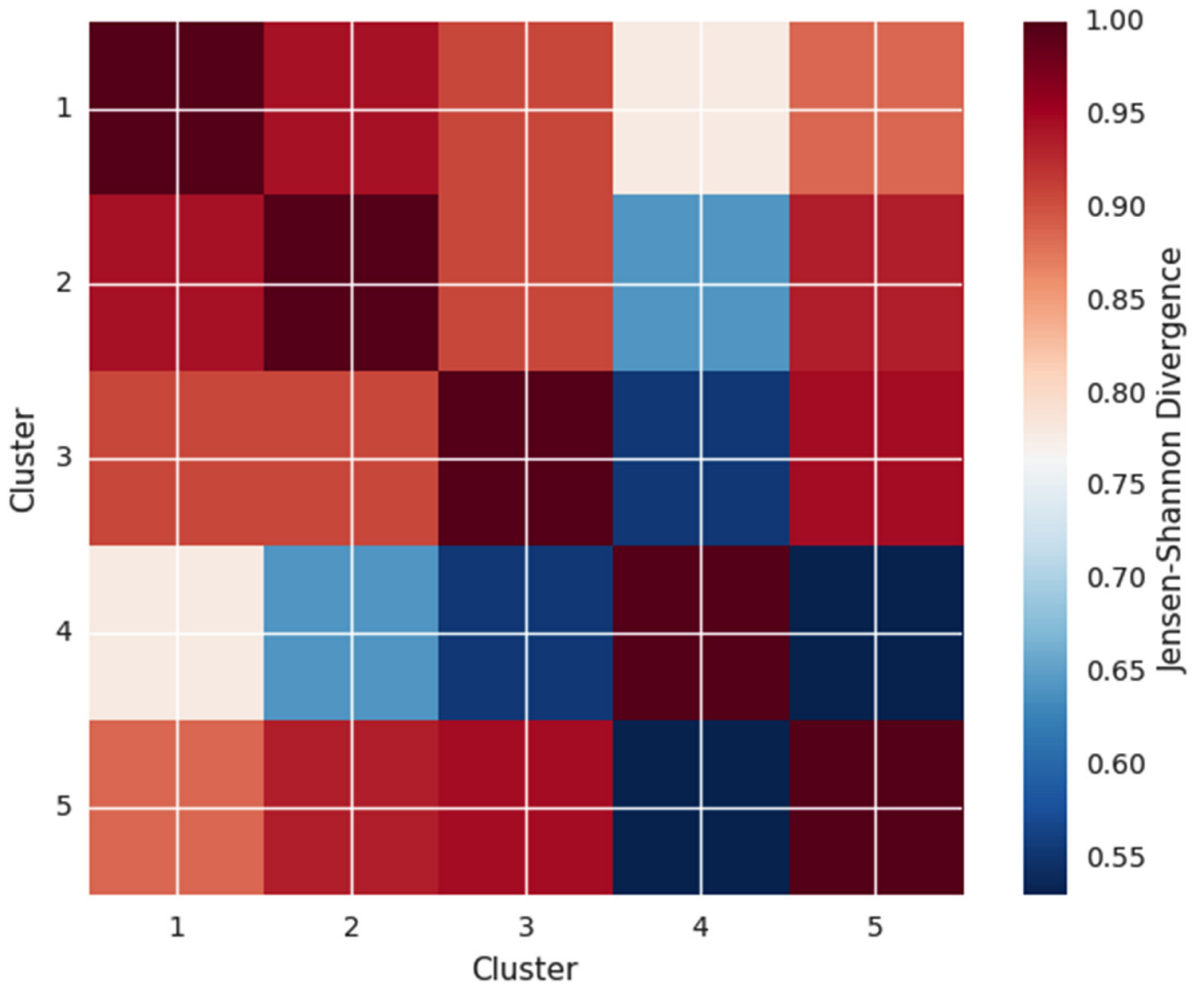
Jensen-Shannon Matrix. Note the large divergence between Clusters 1 and 4, both high marital satisfaction clusters.

Additional evidence for this interpretation comes by aggregating the States distributions within clusters and quantifying the similarity between their probability distributions using the Jensen—Shannon divergence (JSD) method [56, 57]. The JSD method is a symmetrized version of Kullback-Leibler divergence, it has a lower and upper bound, 0 ≤ JSD ≤ 1, and has been shown to be the square of a metric. The JSD comparisons are shown in Fig 6. Note that for Cluster 1, the greatest distributional divergence is with Cluster 4, the other high satisfaction cluster; Clusters 2 and 3, the middle satisfaction groups are very difference from Cluster 4 whereas they are only moderately divergent from Cluster 1. Likewise, Cluster 5, the low satisfaction group, is vastly different than Cluster 4 but not Cluster 1.

### HDP-HSMM Fit

A summary of the results showed minimal differences between the Hamming and the Dynamic Hamming distances, thus for we only report the Hamming distance. With one exception, all best fit combinations were with 20 States. Using the best fitting model criteria, aside from maximum number of States, no other single parameter (e.g., *α* or *γ* in the transition distribution) predominated, either at the couple level or within Cluster.

**Fig 7:**
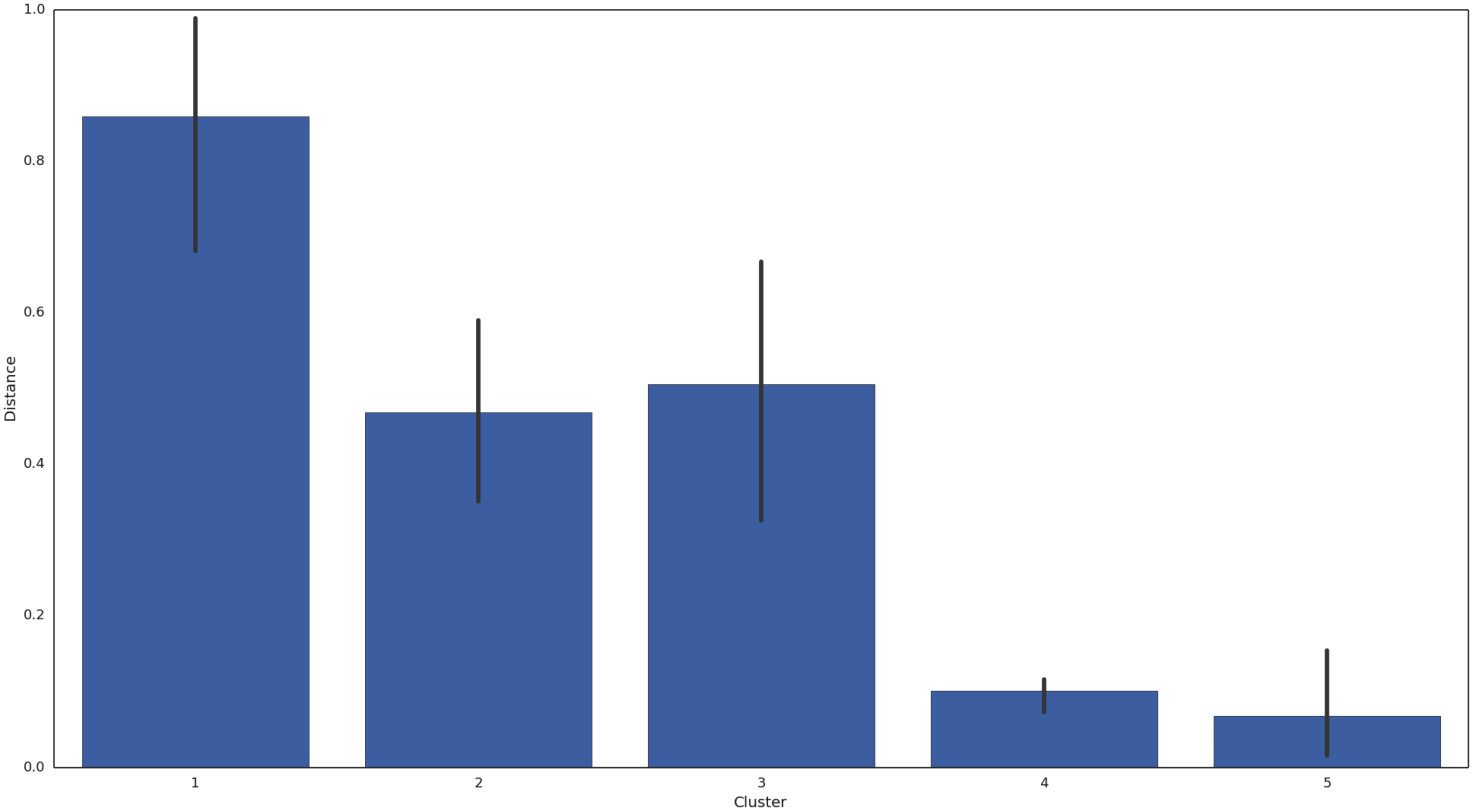
Estimated Hamming Distance Between Simulated and Realized Data by Cluster. Vertical bars reflect the Standard Error.

One clear pattern emerged for the Distance estimates: Cluster types clearly determine the magnitude of the of the average distance between realized and simulated data. As shown in Fig 7, the low satisfaction couples of Cluster 5 and the traditional high satisfied couples of Cluster 4 showed the best simulation estimates; conversely Cluster 1—the highly satisfied but negativity laced couples showed substantially poorer fit. As expected, Cluster 2 and 3, those with the moderate satisfaction levels showed estimates between the extremes. Although Cluster 1 and Cluster 4 consist of highly satisfied couples, Cluster 1 distance estimates were almost 7 times greater than Cluster 4 and 9.15 times greater than Cluster 5.

As expected from Fig 2, MAT level and Distance are correlated (*r* = .56), but this relationship is mitigated by the effects of Cluster 1; a clearer interpretation emerges using the correlation between Distance and Entropy (*r* = .8). It appears that States distribution and structural variability better predicts model fit than couple satisfaction; the best fitting models from Clusters 4 and 5 were from couples showing constrained behavior, either expressing consistently negative (Cluster 5) or consistently positive (Cluster 4) affect. Additionally, we constructed, post analysis, an estimate of entropy rate by Cluster. Entropy rate, an estimate of uncertainty associated with the emergence of symbols in a sequence, also correlated with Distance (*r* = .6), but less than Entropy. This suggests that the overall absence of structure (i.e., Entropy) shows greater association to model fit than entropy rate’s moment-to-moment uncertainty.

### Simulating Dyadic Affective Processes

**Fig 8:**
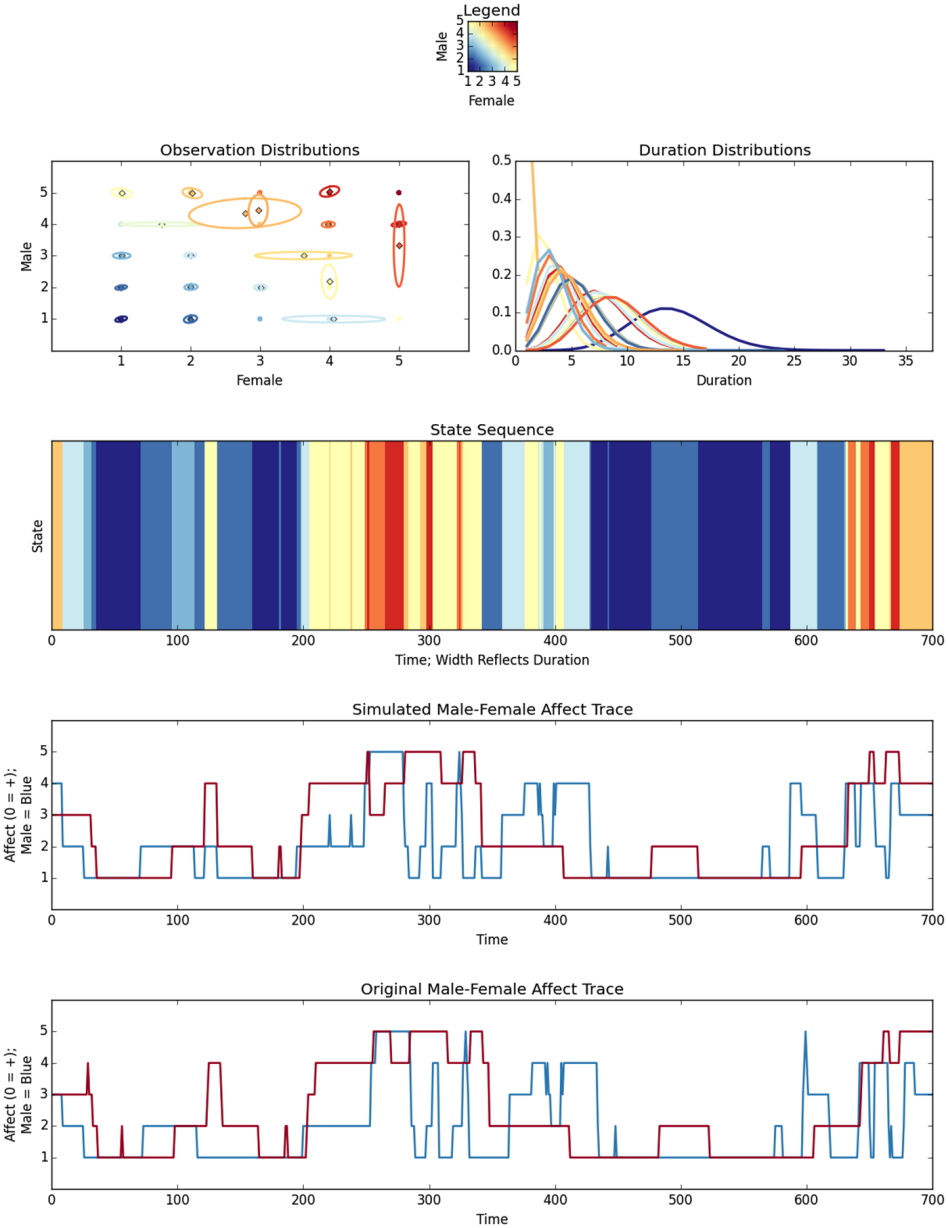
Simulated Distributions Of A Single Dyad From Cluster 1. The upper graphs show a simulated dyadic 2-d Gaussian of male and female joint affect and the simulated duration distribution. The middle graph illustrates the state sequence with durations indicated by band width. Positive affect is indicated by cooler, bluer colors. The final graphs show male-female interactions for simulated and original data, respectively.

**Fig 9:**
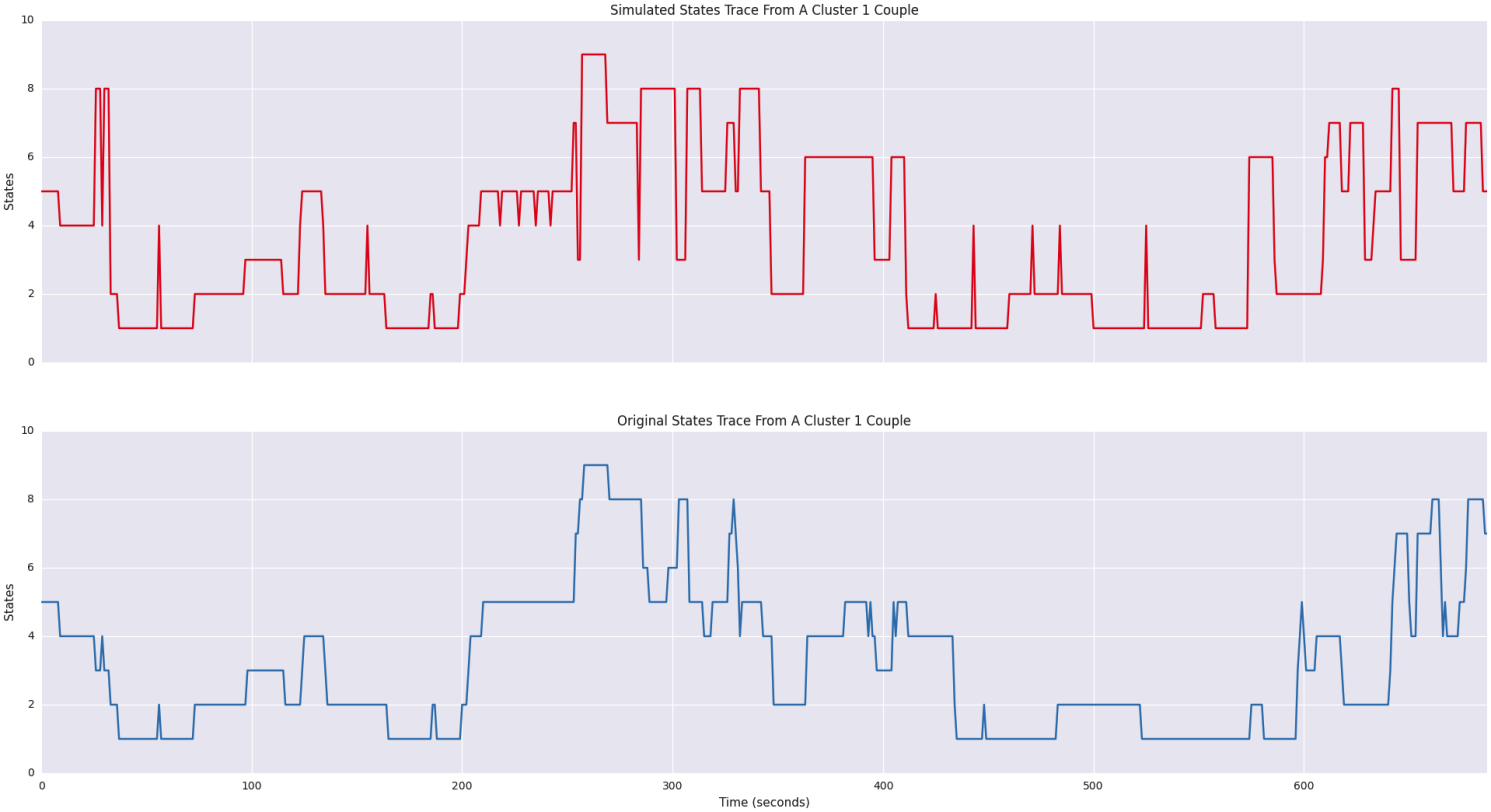
Simulated Sequence Of Dyadic States From Cluster 1. The realized States sequence derived from male-female affect scores is shown below the simulated sequence.

These generative models provide not only a reconstruction of the affect dynamics of a specific interaction but more importantly they provide a means of constructing profiles of hypothetical interactions by actors who like each other in varying degrees. With the HDP-HSMM described herein, modified from code taken from [24], we generate an overall profile showing multiple perspectives of the interactions, illustrating: (1) a 2-dimensional Gaussian distribution reflecting interactant affect; (2) the duration distribution; (3) a sequence and duration profile; (4) a trace of the simulated data; and (5) if applicable, a comparable male-female trace of the realized data. Thus, for example, we can generate an aggregate profile for each Cluster type that reflects the expected behavior from a couple drawn from the Cluster. In Fig 8, we illustrate, a comparison between a simulated distressed couple and their comparable trace from realized data in Cluster 1; additionally, Fig 9 shows a couple States profile from Cluster 1, consisting of highly satisfied couples that express higher than expected levels of negativity. Even this poorest fitting group generate realistic fits to the realized data.

**Fig 10:**
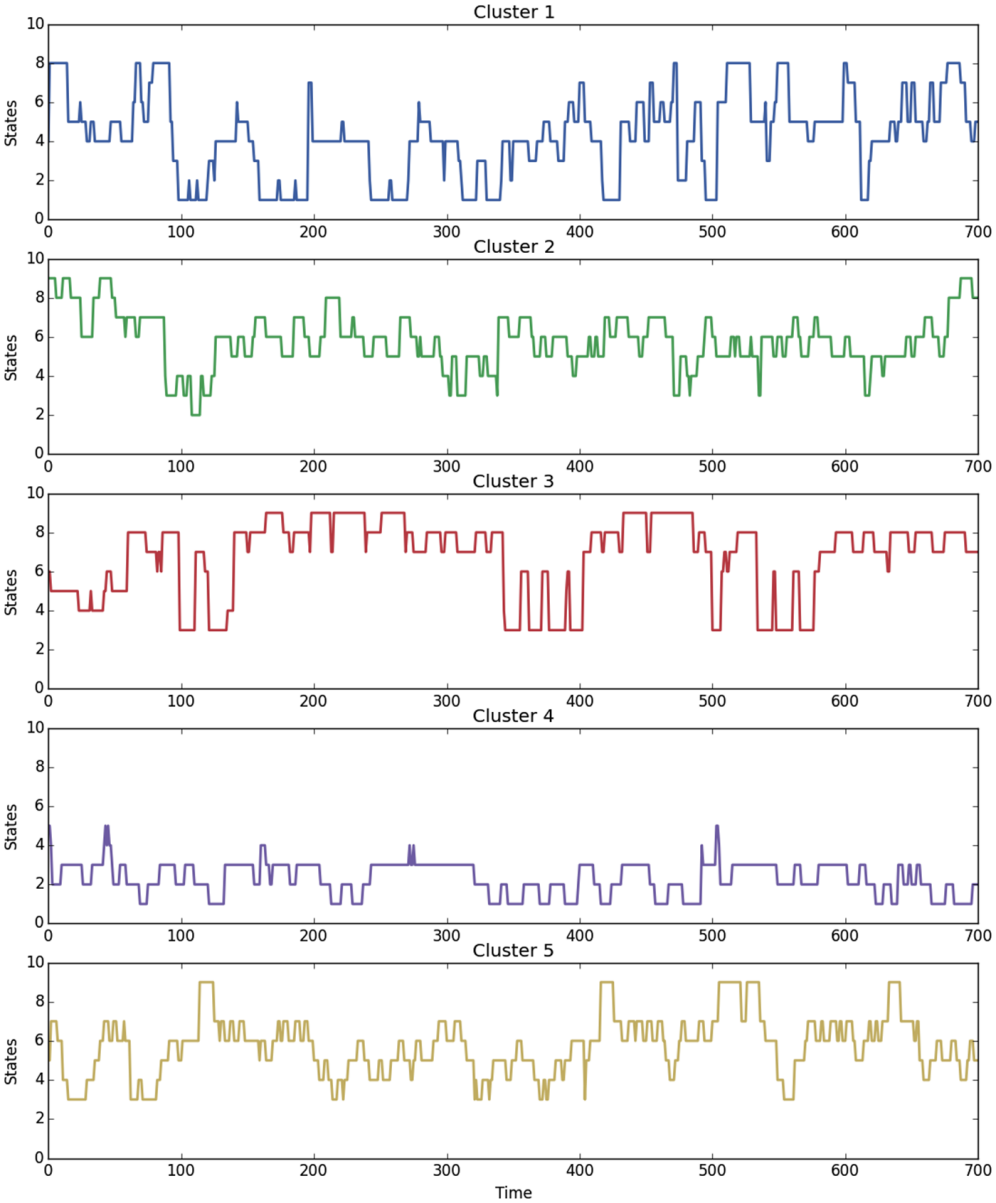
Comparison Of Simulated States Sequences By Cluster. Simulated Clusters show clear States differences consistent with the realized data.

Thus each HDP-HSMM used in this study generates a model reflecting state transitions with expected durations (i.e., *λ* from the Poisson) for each generated state; a draw from each along with the generated transition matrix is then used to generate a plausible sequence of interaction. Likewise, in aggregate, a generic sequence is produced that represents the Cluster tendencies. This potential is illustrated by generating a trace for each cluster type as shown in Fig 10); the sensitivity of interactant relationship satisfaction is evident by the disparate trace patterns. Note especially the contrasting profiles of the highly nondistressed Clusters 1 and 4. Affect in Cluster 4 is positive and constrained whereas Cluster 1 shows moderate affect and high variability. Conversely Cluster 5, the highly distressed group, generates constrained negative affect, consistent with the existing literature [34].

## Discussion

We illustrate how contemporary Bayesian extensions of traditional HMMs can generate realistic multidimensional (i.e., dyadic) interactions. These new methods add realism by incorporating state durations along with the ability to model a countably infinite number of states. These HDP-HSMMs permit investigators to model complex social processes that can vary substantially as a function of the relationship quality of the interactants. Our current study had two objectives: First, we sought to classify dyad dynamics, using self-report affect, into homogeneous groups beyond what had been obtained in the existing literature in this area; Second, we sought to build generative models of these dynamics using contemporary Bayesian machine learning techniques.

Relative to the first objective, historically the method of classifying couple dynamics came from a simple measure of self-report marital satisfaction; our initial attempt to produce homogeneous groups based this single measure were equally inadequate—within group heterogeneity prevented us from building a generic model for each group. Next, using a multi-dimensional feature set, cluster analysis generated five clusters of dyadic interaction styles. Roughly, they broke out into 2 mid-satisfaction level clusters, 1 low satisfaction cluster, and most importantly, 2 high satisfaction clusters—one was typical of the literature—interaction characterized by positive interaction—while the other contained unexpectedly greater variability of affect including high rates of negativity.

Next we used realized data extracted from each cluster to build a HDP-HSMM. These generative models provided a simulation of the captured data as well as a generic model of the dyadic interactions observed in the cluster. Not surprisingly, clusters having greater entropy fit less well than lower entropy clusters, as measured by their Hamming distance. This suggests that affect variability associated with specific satisfaction types influences model fit. Couples with constrained affect—low and traditional high marital satisfaction—mapped very well with the models. Those in the mid-range of MAT scores (e.g., 90-110) produced mid-range fit values, whereas the high-satisfied couples with moderate to high negativity still generated a good model (Cluster 1; see Fig 8), but their fit was the least accurate among all clusters.

These results have a clear implication for subsequent work in Affective Computing—among some actors in an intimate relationship, even those reporting high levels of attraction or satisfaction, sometimes generate elevated and variable levels of negativity. Prior research in the area of couple dynamics report, or assumed, that highly satisfied couples were generally homogeneous and characterized by consistent and high levels of positive behavior and affect. This assumption is inconsistent with the clusters derived from the current data. Although some high satisfied couples were consistently positive (Cluster 4), just as distressed couples were consistently negative (Cluster 5), a sub-group of high satisfied couples also exhibited high level of negativity (Cluster 1). This last group is the most scientifically intriguing: How do couples reporting moderate to high levels of negative affect sustain a satisfying relationship? Its apparent that model fit is not simply a function of affect level but more subtlety the combination of affect and its variability. We assume that some pattern or patterns of variability are associated with each Cluster type—thus the recognition problem becomes an affect * variability categorization task. We are examining that now.

The HDP-HSMM effectively reproduced the realized data across all cluster types, even those with greater affect variability (e.g., Clusters 1, 2, 3). These generative models demonstrated it is possible to take multi-dimensional data reflecting social processes and reasonably recreate complex interactions. To our knowledge this is the first study to demonstrate the applicability of this hierarchical Bayesian technique to social dynamics. Not surprisingly, our results suggest that well-behaved data are easier to model, yet even the poorer fitting models did an acceptable job of capturing duration sensitive state transition dynamics.

## Supporting Information

S1 Appendix Methodology. (PDF)

S1 Text Sample Characteristics. (PDF)

## References

[1] Kiecolt-Glaser JK, Newton TL. Marriage and health: His and hers. Psychological Bulletin. 2001;127(4):472–503. 11439708 10.1037/0033-2909.127.4.472

[2] Kiecolt-Glaser JK, Loving TJ, Stowell JR, Malarkey WB, Lemeshow S, Dickinson SL, et al Hostile Marital Interactions, Proinflammatory Cytokine Production, and Wound Healing. Arch Gen Psychiatry. 2005;62(12):1377–1384. 10.1001/archpsyc.62.12.1377 16330726

[3] Fagundes CP, Bennett JM, Derry HM, Kiecolt-Glaser JK. Relationships and Inflammation across the Lifespan: Social Developmental Pathways to Disease. Social And Personality Psychology Compass. 2011;5(11):891–903. 10.1111/j.1751-9004.2011.00392.x 22125580 PMC3223962

[4] Howe GW, Dagne G, Brown CH. Multilevel Methods for Modeling Observed Sequences of Family Interaction. Journal of Family Psychology. 2005;19(1):72–85. 10.1037/0893-3200.19.1.72 15796654

[5] Bradbury TN, Lavner JA. How Can We Improve Preventive and Educational Interventions for Intimate Relationships? Behavior Therapy. 2012;43(1):113–122. 22304883 10.1016/j.beth.2011.02.008

[6] Dagne GA, Snyder J. Bayesian hierarchical duration model for repeated events: an application to behavioral observations. Journal of Applied Statistics. 2009;36(11):1267–1279. 10.1080/02664760802587032 20209032 PMC2832316

[7] Gottman JM, Roy A. Sequential analysis. New York: Cambridge University Press; 1990.

[8] Griffin WA. Affect Pattern Recognition: Using Discrete Hidden Markov Models to Discriminate Distressed from Nondistressed Couples. Marriage and Family Review. 2002;34(1–2):139–163. 10.1300/J002v34n01_07

[9] Gottman JM, Swanson C, Murray JD. The mathematics of marital conflict: Dynamic mathematical nonlinear modeling of newlywed marital interaction. Journal of Family Psychology. 1999;13:3–19. 10.1037/0893-3200.13.1.3

[10] Picard R. Affective Computing. Cambridge, MA:MIT Press; 2000.

[11] Scherer KR, Banziger T, Roesch E. A Blueprint for Affective Computing: A sourcebook and manual: Oxford University Press. Oxford University Press; 2010.

[12] Calvo RA, D’Mello S. Affect Detection: An Interdisciplinary Review of Models, Methods, and Their Applications. Affective Computing, IEEE Transactions on. 2010;1(1):18–37. 10.1109/T-AFFC.2010.1

[13] Dunson DB. Bayesian dynamic modeling of latent trait distributions. Biostatistics. 2006;7(4):551–568. 10.1093/biostatistics/kxj025 16488893

[14] Gershman SJ, Blei DM. A tutorial on Bayesian nonparametric models. Journal of Mathematical Psychology. 2012;56(1):1–12. 10.1016/j.jmp.2011.08.004

[15] Teh YW, Jordan MI, Beal MJ, Blei DM. Hierarchical Dirichlet Processes. Journal of the American Statistical Association. 2006;101(476):1566–1581. 10.1198/016214506000000302

[16] Emonet R, Varadarajan J, Odobez J. Extracting and locating temporal motifs in video scenes using a hierarchical non parametric Bayesian model. In: Computer Vision and Pattern Recognition (CVPR), 2011 IEEE Conference on; 2011. p. 3233–3240.

[17] Beal MJ, Ghahramani Z, Rasmussen CE. The Infinite Hidden Markov Model In: Dietterich T, Becker S, Ghahramani Z, Pres MIT, editors. In Advances in Neural Information Processing Systems. vol. 14 MIT Press; 2002 p. 577–584.

[18] Heller KA, Teh YW, Gorur D. Infinite Hierarchical Hidden Markov Models. In: Dyk, DV, Welling, M, editors. Proceedings of the Twelfth International Conference on Artificial Intelligence and Statistics (AISTATS-09). vol. 5. Journal of Machine Learning Research—Proceedings Track; 2009. p. 224–231.

[19] Teh YW, Jordan MI. Hierarchical Bayesian Nonparametric Models with Applications In: Hjort N, Holmes C, Müller P, Walker S, editors. Bayesian Nonparametrics: Principles and Practice. Cambridge University Press; 2010 p. 158–207.

[20] Rabiner LR. A Tutorial on Hidden Markov Models and Selected Applications in Speech Recognition. Proc of the IEEE. 1989;77(2):257–286. 10.1109/5.18626

[21] Yoon BJ. Hidden Markov Models and their Applications in Biological Sequence Analysis. Current Genomics. 2009;10(6):402–415. 10.2174/138920209789177575 20190955 PMC2766791

[22] Kehagias A. A hidden Markov model segmentation procedure for hydrological and environmental time series. Stochastic Environmental Research and Risk Assessment. 2004;18(2):117–130. 10.1007/s00477-003-0145-5

[23] Fox EB, Sudderth EB, Jordan MI, Willsky AS. An HDP-HMM for systems with state persistence. In: ICML’08: Proceedings of the 25th international conference on Machine learning. ACM; 2008. p. 312–319.

[24] Johnson MJ, Willsky AS. Bayesian Nonparametric Hidden Semi-Markov Models. Journal of Machine Learning Research. 2013;14:673–701.

[25] Yu S. Hidden semi-Markov models. Artificial Intelligence. 2010;174(2):215–243. 10.1016/j.artint.2009.11.011

[26] Ren L, Dunson DB, Carin L. The Dynamic Hierarchical Dirichlet Process. In: Proceedings of the 25th International Conference on Machine Learning. ICML’08. New York, NY, USA: ACM; 2008. p. 824–831.

[27] Blei D, Lafferty J. Dynamic topic models. In: ICML’06: Proceedings of the 23rd International Conference on Machine Learning. ACM; 2006.

[28] Elzinga CH. Distance, Similarity and Sequence Comparison In: Blanchard P, Bühlmann F, Gauthier JA, editors. Advances in Sequence Analysis: Theory, Method, Applications. vol. 2 of Life Course Research and Social Policies. Springer International Publishing; 2014 p. 51–73.

[29] Studer M, Ritschard G, Gabadinho A, Müller NS. Discrepancy Analysis of State Sequences. Sociological Methods & Research. 2011;40(3):471–510. 10.1177/0049124111415372

[30] Locke HJ, Wallace MK. Short marital adjustment prediction tests: Their reliability and validity. Marriage and Family Living. 1959;21:251–255. 10.2307/348022

[31] Griffin WA. Transitions from negative affect during marital interaction: Husband and wife differences. Journal of Family Psychology. 1993;6(3):230–244. 10.1037/0893-3200.6.3.230

[32] Gunnell GA. Correspondence Between Speaker Affect and Listener Nonverbal Behavior Among Married Couples. M.Sc. Thesis, Arizona State University. Tempe, Arizona State University; 2002.

[33] Gottman JM. Marital Interaction: Experimental investigations New York: Academic Press; 1979.

[34] Heyman RE. Observation of Couple Conflicts: Clinical Assessment Applications, Stubborn Truths, and Shaky Foundations. Psychological Assessment. 2001;13(1):5–35. 10.1037//1040-3590.13.1.5 11281039 PMC1435728

[35] Gottman JM, Levenson RW. A valid procedure for obtaining self-report of affect in marital interaction. Journal of Consulting and Clinical Psychology. 1985;53:151–160. 10.1037/0022-006X.53.2.151 3998244

[36] Hoque ME, McDuff DJ, Picard RW. Exploring Temporal Patterns in Classifying Frustrated and Delighted Smiles. IEEE Transactions on Affective Computing. 2012;3(3):323–334. 10.1109/T-AFFC.2012.11

[37] Weiss RL. Strategic behavioral marital therapy: Toward a model for assessment and intervention In: Vincent JP, editor. Advances in Family Intervention, Assessment and Theory. vol. 1 JAI Press, Greenwich, CT; 1980 p. 229–271.

[38] Shannon C. A Mathematical Theory of Communication. Bell System Technical Journal. 1948;27:379–423, 623–656. 10.1002/j.1538-7305.1948.tb01338.x

[39] Jeffreys H. An Invariant Form for the Prior Probability in Estimation Problems. Proceedings of the Royal Society of London Series A, Mathematical and Physical Sciences. 1946;186(1007):453–461. 10.1098/rspa.1946.0056 20998741

[40] Dasu T, Krishnan S, Venkatasubramanian S, Yi K. An information-theoretic approach to detecting changes in multi-dimensional data streams. In: In Proceedings of the Symposium on the Interface of Statistics, Computing Science, and Applications; 2006.

[41] Krichevsky RE, Trofimov VK. The Performance of Universal Encoding. IEEE Trans Inform. 1981;IT-27:199–207. 10.1109/TIT.1981.1056331

[42] Hausser J, Strimmer K. Entropy inference and the James-Stein estimator, with application to nonlinear gene association networks. Journal of Machine Learning and Research. 2009;10:1469–1484.

[43] Giorgino T. Computing and Visualizing Dynamic Time Warping Alignments in R: The dtw Package. Journal of Statistical Software. 2009;31(7):1–24. 10.18637/jss.v031.i07

[44] Muller M. Information Retrieval for Music and Motion. Springer; 2007.

[45] Crane R, Allgood S, Larson J, Griffin WA. Assessing marital quality with distressed and nondistressed couples: A comparison and equivalency table for three frequently used measures. Journal of Marriage and the Family. 1990;52:87–93. 10.2307/352841

[46] Cohen P. Family measurement techniques: Locke Marital Adjustment Scale and the Dyadic Adjustment Scale. American Journal of Family Therapy. 1985;13:66–71. 10.1080/01926188508251266

[47] L’Abate L, Bagarozzi D. Sourcebook of Marriage and Family Evaluation. New York: Brunner/Mazel; 1993.

[48] Hosking JRM. L-moments: analysis and estimation of distributions using linear combi- nations of order statistic. Journal of the Royal Statistical Society, Series B. 1990;52:105–124.

[49] Sethuraman J. A constructive definition of Dirichlet priors. Statistica Sinica. 1994;4:639–650.

[50] Gabadinho A, Ritschard G, Studer M, Muller NS. Mining sequence data in R with the TraMineR package: A user’s guide; 2010. Available from: http://mephisto.unige.ch/traminer.

[51] Lesnard L. Setting cost in optimal matching to uncover contemporaneous socio-temporal patterns. Sociological Methods & Research. 2010;38:389–419. 10.1177/0049124110362526

[52] Ferreira L, Hitchcock DB. A Comparison of Hierarchical Methods for Clustering Functional Data. Communications in Statistics—Simulation and Computation. 2009;38(9):1925–1949. 10.1080/03610910903168603

[53] Ward JH. Hierarchical Grouping to Optimize an Objective Function. Journal of the American Statistical Association. 1963;58:236–244. 10.1080/01621459.1963.10500845

[54] Kruschke JK. Bayesian data analysis. Wiley Interdisciplinary Reviews: Cognitive Science. 2010;1(5):658–676. 26271651 10.1002/wcs.72

[55] Kruschke JK. Bayesian estimation supersedes the t test. Journal of Experimental Psychology: General. 2013;142(2):573–603. 10.1037/a0029146 22774788

[56] Lin J. Divergence measures based on the Shannon entropy. IEEE Transactions on Information Theory. 1991;37(1):145–151. 10.1109/18.61115

[57] Ré MA, Azad RK. Generalization of Entropy Based Divergence Measures for Symbolic Sequence Analysis. PLoS ONE. 2014;9(4):e93532 10.1371/journal.pone.0093532 24728338 PMC3984095

