# Supplementary material for: Using Bayesian Nonparametric Hidden Semi-Markov Models to Disentangle Affect Processes during Marital Interaction
Source: PLoS One. 2016 May 17;11(5):e0155706. doi: 10.1371/journal.pone.0155706 (PMC4871360; doi:10.1371/journal.pone.0155706)
Supplement: S1 Appendix — (PDF) [file pone.0155706.s001.pdf]

Methodology Overview

Hidden Markov Model

The hidden Markov model (HMM) is a class of doubly stochastic processes based on an underlying, discrete-valued state sequence that is assumed to be Markovian. This underlying stochastic process is not observable, hence the moniker 'hidden', and only knowable via another stochastic process evident as a sequence of observed symbols. Whereas the state generating process is characterized by transition probabilities, the observed process is characterized by a state-dependent probability distribution. Conditioned on this state sequence, the model assumes that the observations are independent, and that each observed variable follows different distributions according to the hidden state of the system, thus implying that some amount of the hidden state is revealed via observation. Used for modeling sequential or time-series data, HMMs have been successfully applied in numerous fields such as speech recognition, computational biology, robot control, machine translation, and finance [3].

More formally, allow $S = \{S_n, n = 1, 2, \ldots\}$ to be a time-homogeneous first-order Markov process and $\Omega = \{\Omega_k, k = 1, 2, \ldots\}$ is a function of S, then S is the hidden Markov process that is observed through Ω, and we can write $\Omega_k = f(S_k)$ for some function f. In this way we can regard S as the *state process* that is hidden and Ω as the *observation process* that can be observed [1].

Using standard notation [1], a Hidden Markov Model is defined as a 5-tuple $(S, \Omega, P, \Phi, \pi)$, where

- $S = \{s_1, s_2, \ldots, s_N\}$ is finite set of N states
- $\Omega = \{o_1, o_2, \ldots, o_M\}$ is a finite set of M possible symbols
- $P = \{p_{ij}\}$ is the set of state-transition probabilities, where p_{ij} is the probability going to state s_j from state s_i
- $\Phi = \{\phi_i(o_k)\}$ are the observation probabilities, where $\phi_i(o_k)$ is the probability of emitting symbol o_k when the system is in state s_i
- $\pi = \{\pi_i\}$ is initial starting state

This 5-tuple $(S, \Omega, P, \Phi, \pi)$ is often abbreviated to $\lambda = (P, \Phi, \pi))$ when the states and emission sequences are understood.

Three fundamental problems for HMMs

To effectively construct a HMM three problems need to be addressed:(1) Evaluation; (2) Decoding; and (3) Learning.

- Evaluation: What is the probability that a particular model produced a particular observation? That is, given a model $\lambda = (P, \Phi, \pi)$ and an observation sequence $O = \upsilon_1, \upsilon_2, \ldots, \upsilon_T$ of length T, where $\upsilon_i \in \Omega$, what is the probability that the model generated the observation sequence; that is, what is $P[O|\lambda]$?
- Decoding: What is the most likely state transition path associated with an observed sequence? That is, again, given a model $\lambda = (P, \Phi, \pi)$, what is the most likely sequence of hidden states that could have generated a given observation sequence, $O = \upsilon_1, \upsilon_2, \ldots, \upsilon_T$ of length T?
- Learning: Estimate the most likely HMM parameters for a given observation sequence; that is, given a set of observation sequences,find the values for λ that maximize $P[O|\lambda]$.

Fortunately, methods are well established for solving each of the aforementioned problems. The evaluation problem is usually solved by the forward and the backward iterative algorithms. Next, the decoding problem is solved by using the Viterbi algorithm, also an iterative algorithm, to fabricate the best path by sequentially considering each observed symbol. Finally,the learning problem is solved by the Baum-Welch algorithm (an Expectation Maximization algorithm), which uses the forward and backward probabilities to update the parameters iteratively. There are numerous excellent references that detail the basic components of the HMM (i.e., [1], [2], [3]) and will not be reviewed in detail here.

Despite its utility and popularity across numerous fields in engineering and science, HMMs have two significant disadvantages: (1) state duration distributions are restricted to a geometric form that is not appropriate for many real-world data, and (2) the number of hidden states must be set a priori so that model complexity is not inferred from data in a Bayesian way. We address the geometric distribution problem first.

Integrating the HMM and State Duration: Hidden Semi-Markov Models

Most real-world sequential phenomena, especially those involving social and natural dynamics, are highly dependent on time-in-state as a critical feature of state change. HMMs implicitly assume that time spent in a given state is distributed according to a geometric distribution. This distribution is memoryless, meaning that, at a given time t, the waiting time for switching from one state to another is independent of state duration or *sojourn time*. Stated differently, hidden Markov models have a constant probability of changing state given survival in the state up to that time. Conversely, in social interaction, an interactant's behavior is not memoryless but rather tightly bound to immediacy, within historical and relationship precedent. Consequently, the HMM generates inadequate temporal structure of dynamic processes.

A generalization of the HMM, the hidden semi-Markov model (HSMM) allows the hidden process to be semi-Markovian; this means that the probability of transitions among the hidden states depends on the amount of time that has elapsed since entry into the current state i [4]. The standard HMM emits one observation per state whereas a HSMM state can emit a sequence of symbols; the symbol sequence length - being its duration d - for each state is determined by some state-specific distribution. This state change process along with its variable length symbol emissions is illustrated in Figure 1.

Aside from some minor modifications in the aforementioned evaluation, decoding, and learning algorithms, the HSMM is structurally similar to the HMM with the additional incorporation of a state duration variable [5]. State duration is a random variable and assumes an integer value in the set $D = \{1, 2, ..., D\}$. We use the random variable D_t to denote the duration of a state that is entered at time t, and we write the probability mass function for the random variable as $p(d_t|x_t = i)$, where x_t is the state sequence with $t \in \{1, \ldots, T\}$. In short, the important difference between HMM and HSMM is that one observation per state is assumed in HMM while in HSMM each state can emit a sequence of observations, where the number of observations produced while in state i is determined by the length of time spent in state i, i.e., the duration d [4].

Integrating the HMM and Infinite States Spaces: HDP-HSMM

As noted above, a shortcoming of the HMM, at least from a Bayesian perspective, is its inability to infer the number of states necessary to model system complexity; more specifically, the state space is created a priori and does not get modified as new data are acquired. In the present study, the problem is straightforward: Find a method for determining the appropriate number of states in the model that is simultaneously

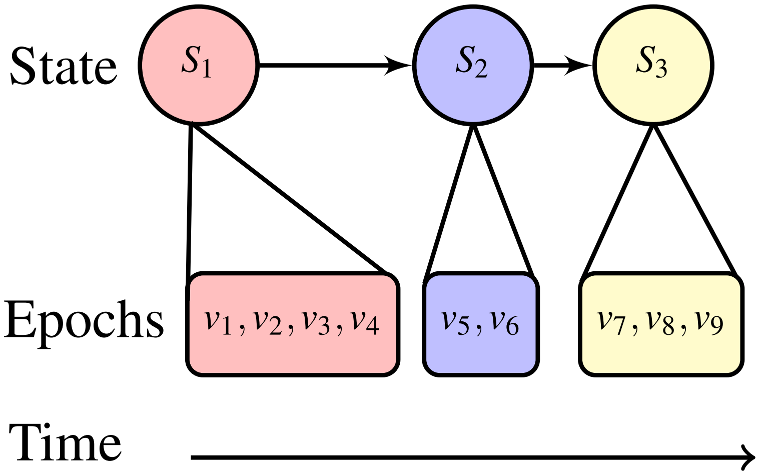

Fig 1. Illustrating HSMM Epoch Strings Across State Changes. Note state S_i changes by color showing variable length durations over the period of observation. Adopted from [1]

sensitive to couple type and allows for within type couple variability. Fortunately within the last decade a clear methodology for this type of problem arose—the Hierarchical Dirichlet Process (HDP) [6], a Bayesian nonparametric approach that estimates how many groups (i.e., states) are needed to model the observed data. Nonparametric implies, not that the model is without parameters, but rather that model parameters change as data additional data are acquired. Within this framework nonparametric models are termed *infinite-dimensional*, technically however, rather than being infinite, they can be evaluated on a finite sample in a manner that uses only a finite subset of the available parameters to explain the sample [7]. In other words, within the context of an HDP-HMM and HDP-HSMM, the number of states is unbounded and allowed to grow with the sequence length—new states are added as model complexity increases.

To understand the Hierarchical Dirichlet Process (HDP), it's necessary to first grasp the concept and relevance of the Dirichlet Process (DP). The Dirichlet process is a stochastic process that defines a probability distribution over infinite-dimensional discrete distributions, meaning that a draw from a DP is itself a distribution. It's typically described as a distribution over probability distributions. A DP has two parameters, a concentration parameter α and the base measure β; the base distribution β is the parameter on which the nonparametric distribution is centered, which can be thought of as the prior; it is similar to the mean of a Gaussian distribution and the concentration parameter α, given as a positive scalar, is analogous to a variance measure and expresses the the strength of belief in β. For small values of α, samples from a DP are constrained to a small number of atoms, larger values generate more dispersion across the distribution. Viewed differently, α is the inverse of the variance. Estimating parameters for the DP is obtainable in multiple ways—as a Stochastic Process [8], a Chinese restaurant process [9], a Pólya urn process, or a Stick Breaking construction [10]; with some slight variation, these are analogous.

The HDP's unique hierarchical arrangement provides mathematical convenience and conserves dimensional spareness, in our associated article this refers to *States*. Essentially the HSMM is a mixture model where each component corresponds to a state of the HSMM. Given a state, the model needs to emit a sequence of observations and advance to the next state. Within this framework, component parameters specify the distributions associated with these stochastic choices; that is, the transition parameters

specify a distribution over states, which is naturally represented with a draw from a DP. The HDP framework, with its base distribution β (i.e., average of state probabilities; derived, in our study, via a stick-breaking process), and the specific transition level distributions, DP(β_i,α) ties together the transition DPs. This enables the transition distributions to have their mass concentrated around a typical set of states, providing the desired bias towards re-using a constrained and consistent set of states [11].

References

1. Ibe OC. Markov processes for stochastic modeling. Elsevier Insights. Amsterdam: Elsevier, 2nd ed.; 2013.
2. Rabiner LR. A Tutorial on Hidden Markov Models and Selected Applications in Speech Recognition. Proc of the IEEE. 1989;77(2): 257–286.
3. Zucchini W, MacDonald IL. Hidden Markov models for time series : an introduction using R. Monographs on statistics and applied probability. Boca Raton: CRC Press; 2009.
4. Yu S. Hidden semi-Markov models. Artificial Intelligence. 2010;174(2): 215 – 243.
5. Guédon Y. Estimating hidden semi-Markov chains from discrete sequences. Journal of Computational and Graphical Statistics. 2003;12: 604-639.
6. Teh YW, Jordan MI, Beal MJ, Blei DM. Hierarchical Dirichlet Processes. Journal of the American Statistical Association. 2006;101(476): 1566–1581.
7. Orbanz P, Teh YW. Bayesian nonparametric models. Encyclopedia of Machine Learning, Springer; 2010.
8. Ferguson TS. A bayesian analysis of some nonparametric problems. The Annals of Statistics. 1973;1: 209–230.
9. Pitman J. Combinatorial stochastic processes. Technical report, Technical Report 621, Dept. Statistics, UC Berkeley, 2002. Lecture notes for St. Flour course.
10. Sethuraman J (1994) A constructive definition of Dirichlet priors. Statistica Sinica. 1994;4: 639-650.
11. Johnson MJ, Willsky AS. Bayesian Nonparametric Hidden Semi-Markov Models. Journal of Machine Learning Research. 2013;14:673–701.
