# Supplementary material for: Using Bayesian Nonparametric Hidden Semi-Markov Models to Disentangle Affect Processes during Marital Interaction
Source: PLoS One. 2016 May 17;11(5):e0155706. doi: 10.1371/journal.pone.0155706 (PMC4871360; doi:10.1371/journal.pone.0155706)
Supplement: S1 Text — (PDF) [file pone.0155706.s002.pdf]

Sample Characteristics

Sample

Participants were young, Caucasian, moderately educated, lower-middle class couples with one child at home. In the distressed group, male ages ranged from 26–49 (μ = 35.64, σ = 6.85) and for females, ages ranged from 24–49 (μ = 34.07, σ = 6.26). In the nondistressed group, male ages ranged from 24–46 (μ = 32.19, σ = 6.78) and for females, ages ranged from 20–44 (μ = 29.75, σ = 6.26). Twenty-four couples were Caucasian, one couple was African-American, and five couples were racially mixed. Three of the mixed race couples were in the distressed group and two mixed race couples were in the nondistressed group. The African-American couple was in the distressed group. On average, participating couples had nearly two years of college education. In the distressed group, husband education scores ranged from 11–20 (μ = 13.93, σ = 2,37) and for wives, education scores ranged from 11–19 (μ = 13.5, σ = 2.21).

In the nondistressed group, husband education scores ranged from 10–19 (μ = 14.0, σ = 2.78) and for wives, education scores ranged from 12–17 (μ = 13.88, σ = 1.75). Distressed couple incomes, in 1993 dollars, ranged from $12,000$–$52,000$ (μ = $29,000$, σ = $13,098$), whereas nondistressed couples incomes ranged from $4,999$–$52,500$ (μ = $24,000$, σ = $13,813$).

The number of children living at home for both distressed and nondistressed couples ranged from 0 to 4, but distressed couples were more likely than nondistressed couples to have a greater number of children living at home (e.g. mean number of children living at home for distressed couples ranged from 0–4 (μ = 1.79, σ= 1.42) and 0–4 (μ = .81, σ = 1.11) for nondistressed couples. Half of the distressed husbands and over one-quarter (.286) of distressed wives had been previously married. Approximately one-third of nondistressed husbands (.313) and nondistressed wives (.375) had been previously married. Mean number of previous marriages for distressed husbands was .86 (range = 0-4; σ = 1.17) and .29 (range = 0-1; σ = .47) for distressed wives. Mean number of previous marriages for nondistressed husbands was .56 (range = 0-4; σ = 1.09) and .44 (range = 0-2; σ = .63) for distressed wives. To determine group comparability, a series of t-tests assessed differences between distressed and nondistressed groups for: age, education, income, current marriage length, and number of children at home. Only number of children living at home was statistically significant between distressed and nondistressed couples ($t(28)$ = 2.10, p $<$ .05). This difference appears to be due, in part, to the relationship between the distressed mothers having been married longer (r = .337) and being slightly older (r = -.336). As expected, there is a moderate correlation between number of children and length of marriage (r = .349).
